# Fixation of a Periprosthetic Intertrochanteric Hip Fracture below a Birmingham Hip Resurfacing

**DOI:** 10.1155/2014/393984

**Published:** 2014-06-05

**Authors:** J. Macdonald, A. Robinson, I. Brown

**Affiliations:** The Ulster Hospital, Dundonald, UK

## Abstract

This case report involves a 56-year-old female (Mrs X) with a traumatic intertrochanteric hip fracture with subtrochanteric extension below a previous Birmingham hip resurfacing. Periprosthetic fractures following hip resurfacing are usually subcapital and treated with a revision or conservative management. We present an unusual surgical problem with an interesting solution stabilising the fracture using a proximal femoral locking compression plate (LCP). Eight months following surgery the patient is able to walk pain free and there is good fixation and stability.

## 1. Presentation

**Figure 1:**
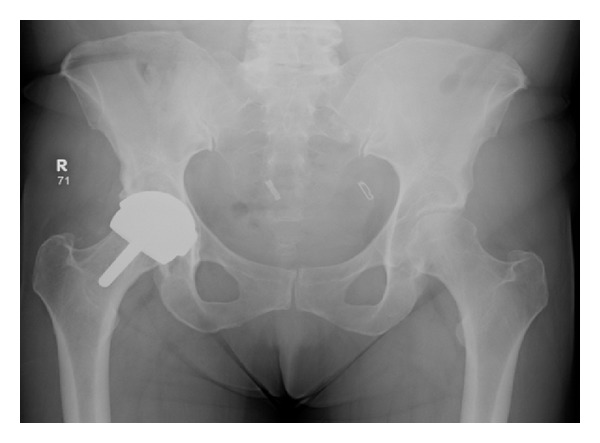
Pelvis radiograph from 2009.

**Figure 2:**
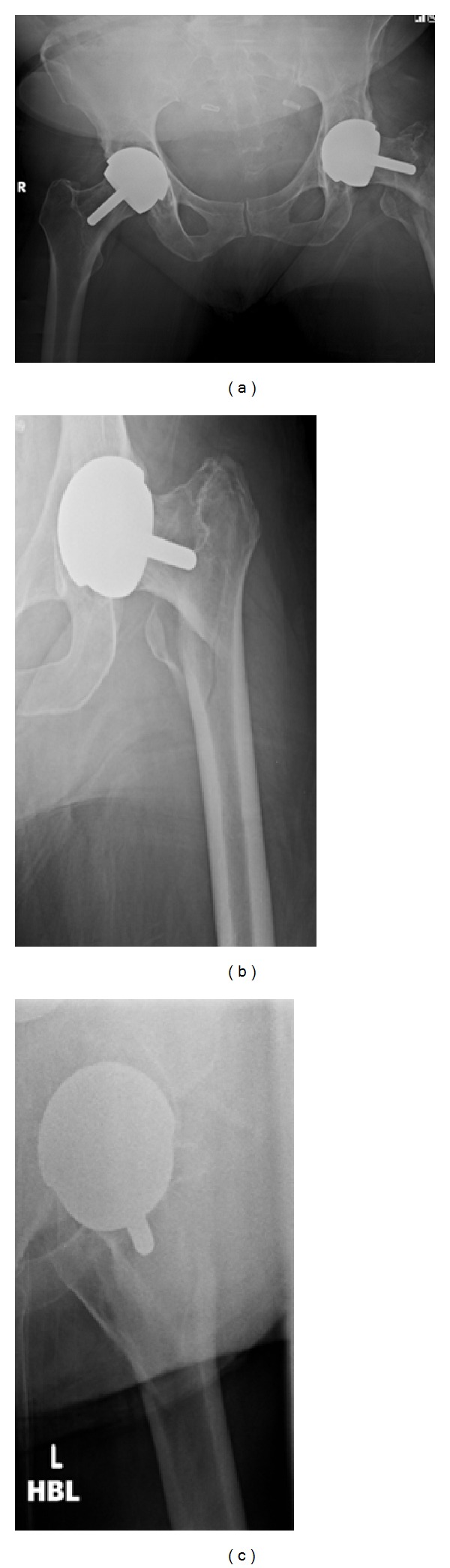
(a) and (b), AP views. (c) Lateral view.

A 56-year-old lady presented to hospital after a fall onto her left hand side, after which she was unable to weight bear. On examination her leg was shortened, externally rotated, and neurovascularly intact. There was a history of previous bilateral Birmingham hip resurfacing (Smith & Nephew) with the left side five years prior to admission and the right side one year before. Other past medical history included, osteoarthritis affecting hands, hips, knees and spine, as well as hypertension and diet controlled type 2 DM. There had also been a recent history of increased alcohol consumption. A pelvic X-ray taken in 2007 demonstrated significant bilateral hip osteoarthritis.  Figure 1 shows a radiograph from 2009 after her right hip resurfacing and prior to surgery on the left.  Figure 2 demonstrates the injury sustained in early December 2012.

## 2. Management

The patient received initial standard investigation and management for her hip fracture. The case was discussed and X-rays were reviewed at the weekly meeting with multiple consultants present including a hip revision surgeon. Due to the patients age it was felt that preservation of bone stock and avoiding revision of the hip resurfacing (which had been functioning well) was preferable.

**Figure 3:**
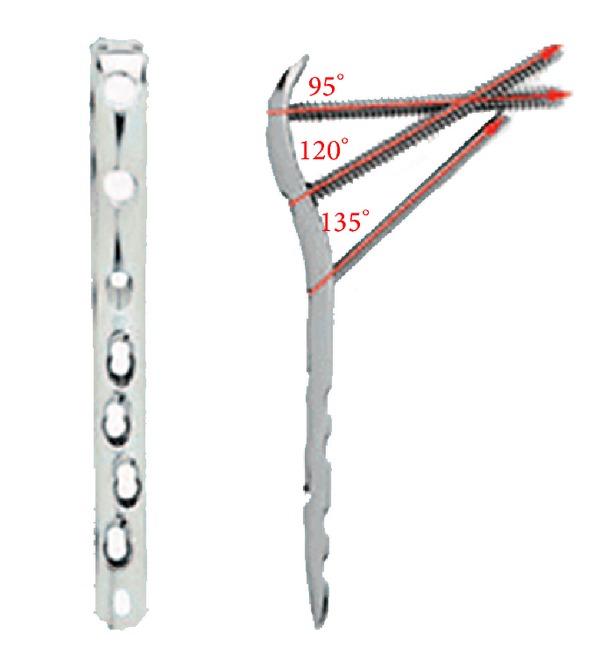
LCP locking plate.

On discussion with the patient and reviewing what equipment was available locally it was decided that using a LCP proximal femoral locking plate (Synthes) would provide the needed fixation without interfering with the current prosthesis. See Figure 3.

## 3. Surgical Technique

Operation performed: open reduction internal fixation of left hip periprosthetic fracture with cerclage cable augmentation.

Operative findings: Previous Birmingham hip resurfacing with an intertrochanteric fracture with subthrochanteric extension.

**Figure 4:**
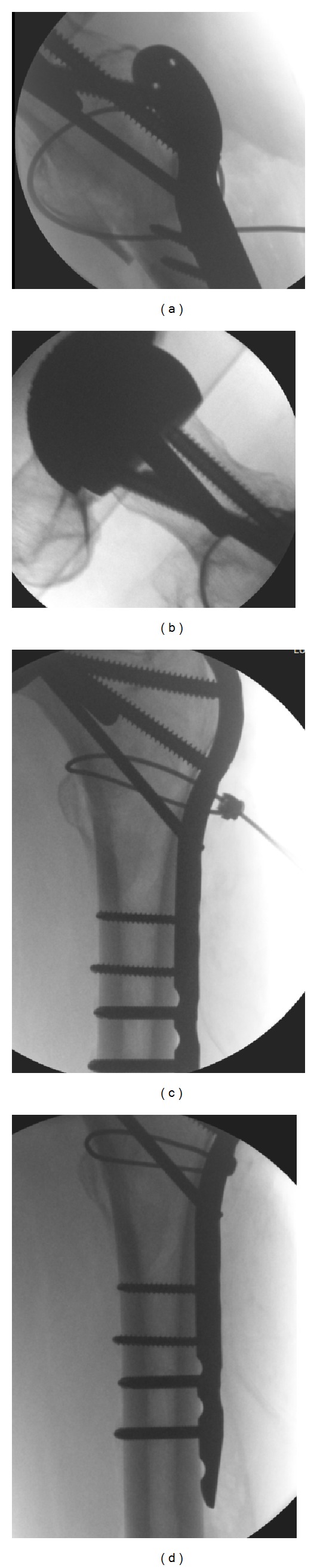
(a)–(d) Showing intraoperative screening.

Operative procedure: Mrs X was positioned on the traction table and closed reduction was achieved. Through a lateral approach a LCP proximal femoral plate was placed. A combination of locking and nonlocking screws was used with two passing posterior and one passing anterior to the stem of the Birmingham hip resurfacing prostehesis. Finally, a cerclage wire reduced and held the lesser trochanter fragment.  Figure 4 shows the intraoperative screening images.

Post-operative plan: Minimal weight bearing for six weeks and a clinic review at this point.

## 4. Outcome

Postoperatively Mrs X developed a chest infection and was treated with IV antibiotics and recovered well; she was discharged home 10 days post-operatively. She was to remain light partial weight bearing for six weeks until her clinic review.

**Figure 5:**
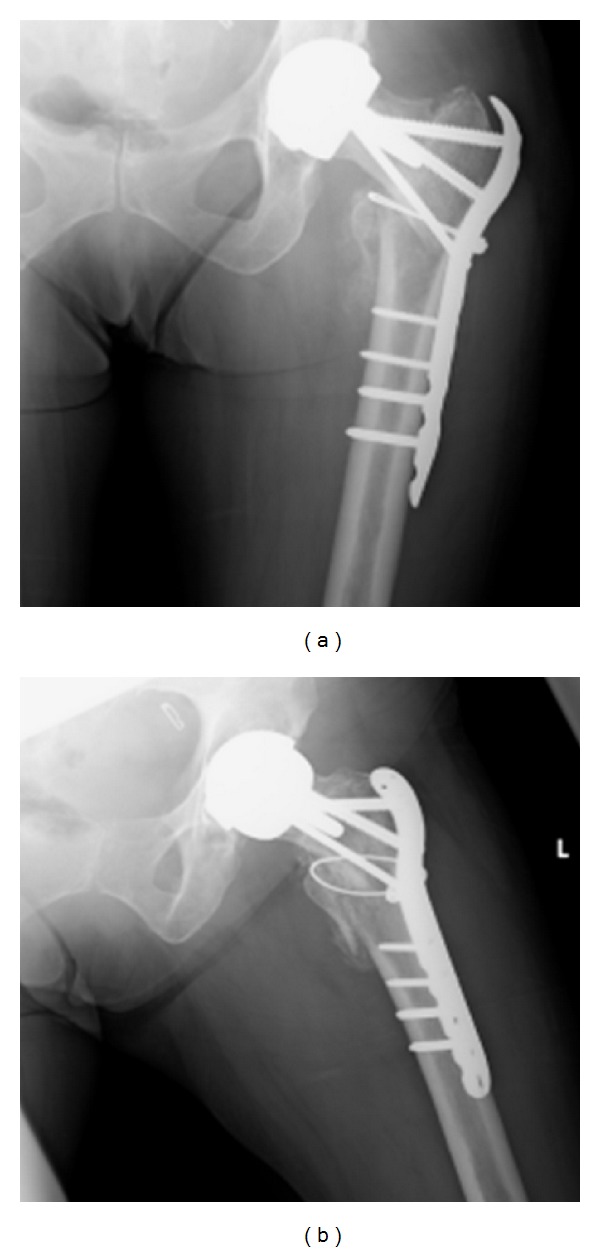
(a)-(b) Images taken at 6-week postoperation review.

Mrs. X was reviewed six weeks post-operatively and it was noted that her fracture had collapsed slightly and the hip had slipped into a more varus position (see Figure 5). At this stage light partial weight bearing was continued and a review was arranged for a further six weeks.

**Figure 6:**
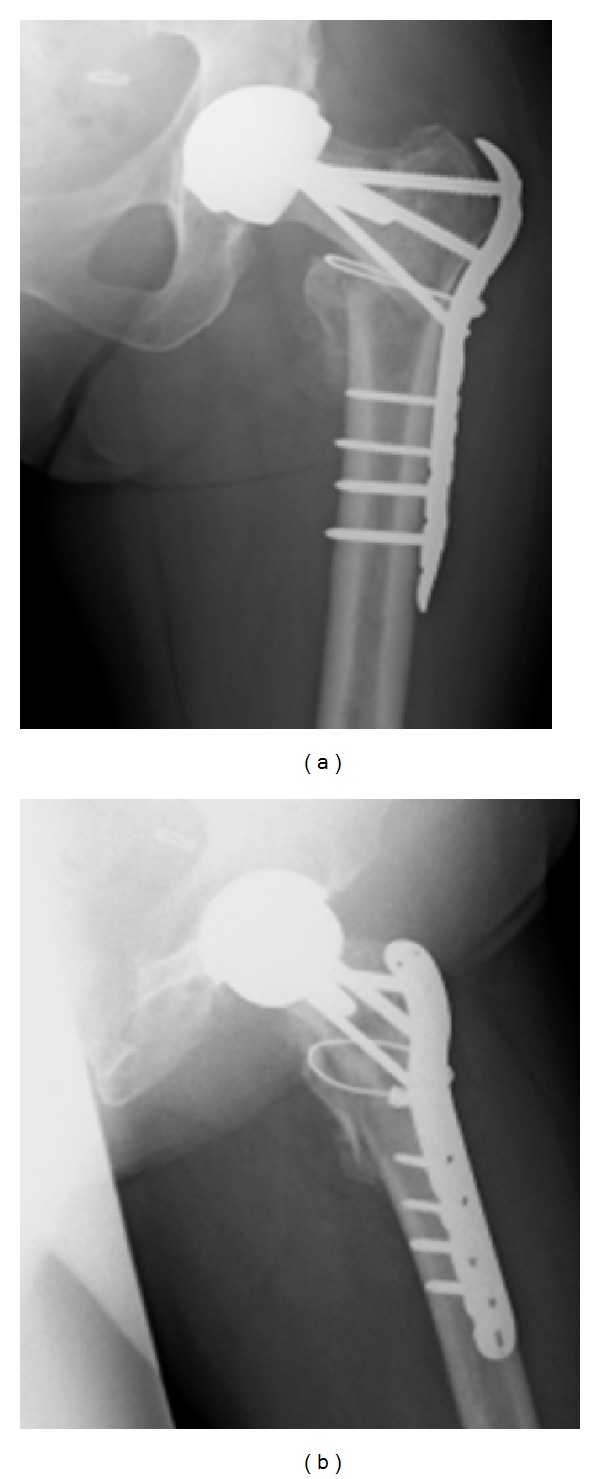
(a)-(b) Radiographs from 12 weeks after operation.

At 12 weeks post-operatively, it was noted that there was good callus formation at the fracture site and that the fracture appeared to have stabilised, with no evidence of metalwork failure (see Figure 6). Mrs X was advised to wean herself off crutches and was referred for physiotherapy.

**Figure 7:**
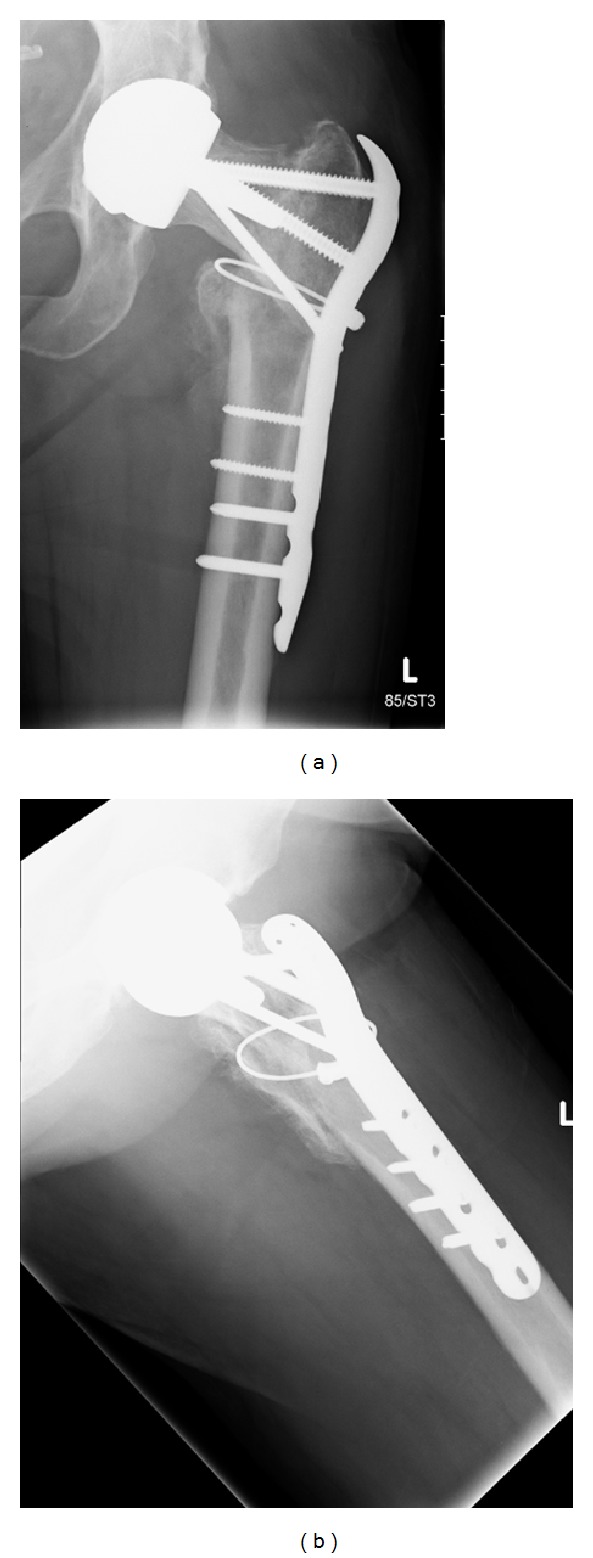
Showing an AP and a lateral view 4 months from surgery.

At a further review 4 months post-operatively, X-rays demonstrated ongoing healing; however there continued to be a small area in the superior neck which was not fully consolidated (see Figure 7). Mrs X was fully weight bearing and allowed to return to aquarobics.

**Figure 8:**
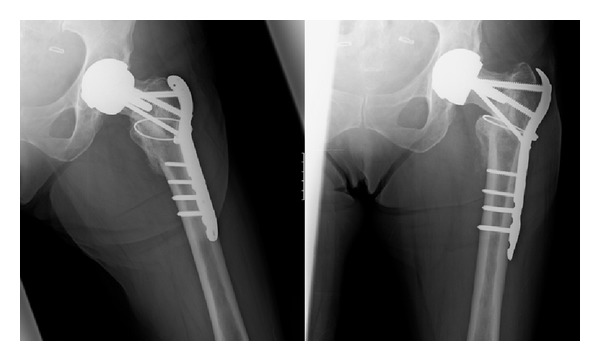
AP views, 8 months from surgery.

At a review eight months from surgery it was noted that whilst the fracture had slipped into varus soon after surgery, subsequent comparison X-rays showed there was no further slip with good callus formation (see Figure 8). Mrs X was able to walk with no pain and used a stick for stability. She complained of some tenderness over the lateral wound with the metal work underlying. At this stage it was thought there was good fixation and stability.

During her rehabilitation, Mrs X was also referred for review by the specialist nurse-led osteoporosis team. She had a Dexa scan preformed which showed a T score of 1.6 in the lumber spine and a score of 1.2 at the distal radius, demonstrating no increased risk of osteoporosis. Despite this, she was commenced on supplements due to her recent atypical fracture.

## 5. Discussion

Fracture of the femoral neck is a recognised complication following Birmingham hip resurfacing [1–3]. Fractures around hip resurfacing prosthesis have two modes of presentation, atraumatic and traumatic fractures [4]. The most common fracture pattern for both modes is a subcapital fracture; any other type of fracture is rarely reported. The main stay of management has been with a revision to a total hip prosthesis, however non operative management has also been found to be effective [3–6]. There is little literature available on internal fixation of the fracture site [4, 7]. In the available literature we found two other cases regarding a similar fracture pattern below a Birmingham hip resurfacing. Each case used a femoral locking plate with good effect to treat the fracture.

We present a 56-year-old female with a traumatic intertrochanteric hip fracture with subtrochanteric extension below a previous Birmingham hip resurfacing.

For this case conservative management was not considered due to the fracture displacement. Conservation of bone stock with a view to possibly requiring later revision surgery was felt to be the best option for this young lady. On discussion with the patient a decision was made to preserve the current implant, which had been performing well, and use an internal fixation device.

**Figure 9:**
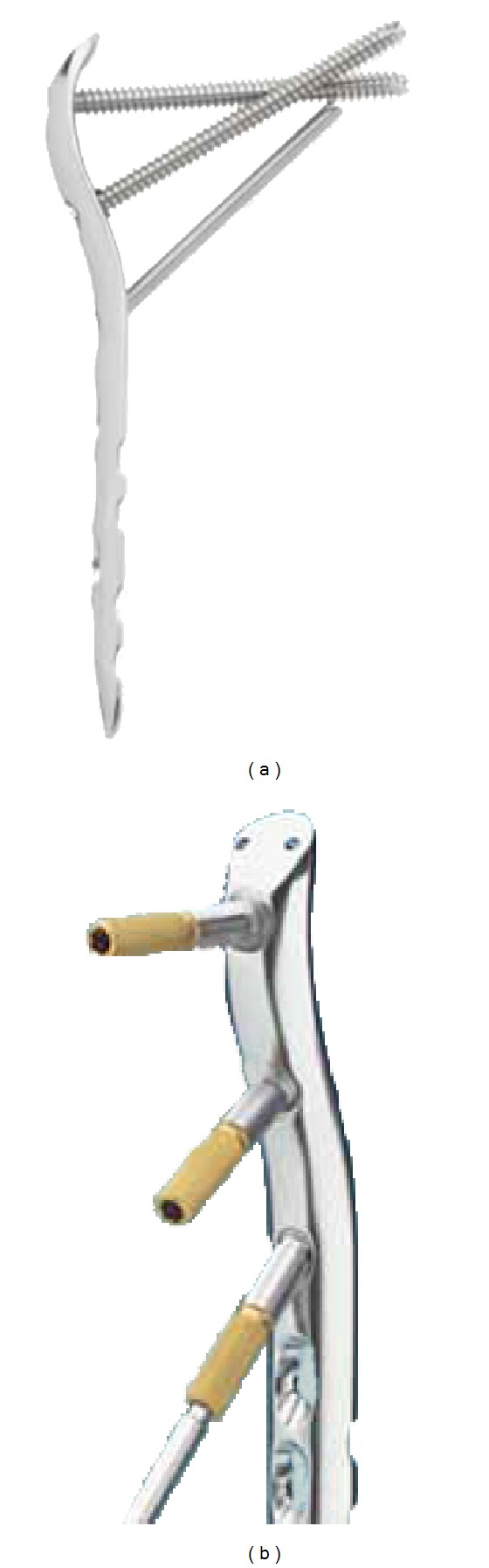
Positions of the screw holes with drill guides.

The main concern with using the internal fixation device was the placement of the screws and the stem of the current hip prosthesis. A decision was made to utilise a Synthes LCP proximal femoral plate [8]. This plate was selected to allow anatomical reduction and fixation with screws at angles that would not disrupt the pre-existing prosthesis. The plate design makes it possible to pass the screws both anterior and posterior to the stem, with the first and third screws passing posterior and the second screw passing anterior (Figure 9).

With regard to the fracture slipping into a more varus position; it was discussed that additional screws may add stability but would be difficult to position appropriately. The team felt that if a similar position was obtained intraoperatively in future, additional screws would not be used as we would expect the fracture to heal in that position. A longer period of protected weight bearing until radiological evidence of union (either X-rays or metal artefact reduction CT) may be of benefit.

## 6. Conclusion

In conclusion, it has been demonstrated that surgical fixation of a fracture below Birmingham hip prosthesis can be adequately carried out with good functional recovery. Factors such as fracture configuration, mobility, age, bone quality and likelihood of requiring later revision surgery need to be taken into account. We would recommend consideration of the LCP proximal femoral plate for fixation of rarely reported extracapsular fractures below hip resurfacing prostheses.
